# Disproportionality Analysis From World Health Organization Data on Semaglutide, Liraglutide, and Suicidality

**DOI:** 10.1001/jamanetworkopen.2024.23385

**Published:** 2024-08-20

**Authors:** Georgios Schoretsanitis, Stefan Weiler, Corrado Barbui, Emanuel Raschi, Chiara Gastaldon

**Affiliations:** 1 The Zucker Hillside Hospital, Department of Psychiatry, Northwell Health, Glen Oaks, New York; 2 Department of Psychiatry, Zucker School of Medicine at Northwell/Hofstra, Hempstead, Glen Oaks, New York; 3 Department of Psychiatry, Psychotherapy and Psychosomatics, Hospital of Psychiatry, University of Zurich, Zurich, Switzerland; 4 Institute of Pharmaceutical Sciences, Department of Chemistry and Applied Biosciences, ETH Zurich, Zurich, Switzerland; 5 Institute of Primary Care, University of Zurich and University Hospital Zurich, Zurich, Switzerland; 6 WHO Collaborating Centre for Research and Training in Mental Health and Service Evaluation, Department of Neuroscience, Biomedicine and Movement Sciences, Section of Psychiatry, University of Verona, Verona, Italy; 7 Pharmacology Unit, Department of Medical and Surgical Sciences, University of Bologna, Bologna, Italy; 8 Institute of Social and Preventive Medicine, University of Bern, Bern, Switzerland

## Abstract

**Importance:**

Glucagon-like peptide-1 receptor agonists (GLP-1 RAs) have gained use primarily due to their weight-reduction effects, although a regulatory review was undertaken for potential suicidality concern.

**Objectives:**

To evaluate potential signals for suicidal and self-injurious adverse drug reactions (ADRs) associated with the GLP-1 RAs semaglutide and liraglutide.

**Design, Setting, and Participants:**

Disproportionality analysis through the case-control design using the World Health Organization (WHO) global database of suspected ADRs. Participants were clinical patients worldwide experiencing an ADR suspectedly attributable to semaglutide or liraglutide in the database from inception to August 30, 2023. Data were analyzed from September to December 2023.

**Exposure:**

Treatment with semaglutide or liraglutide regardless of indication or treatment duration.

**Main Outcomes and Measures:**

Reporting odds ratio (ROR) and the bayesian information component (IC) with 95% CIs were calculated as measures of disproportionate reporting of suicidal and self-injurious ADRs associated with semaglutide and liraglutide compared with all other medications. Sensitivity analyses were conducted including patients with coreported use of antidepressants and benzodiazepines and using dapagliflozin, metformin, and orlistat as comparators. A disproportionality signal was considered when the lower limits of the ROR and IC were above 1 and 0, respectively.

**Results:**

A total of 107 (median [IQR] age 48 [40-56] years; 59 female patients [55%]) and 162 (median [IQR] age 47 [38-60] years; 100 female patients [61%]) cases of suicidal and/or self-injurious ADRs were reported between November 2000 and August 2023 with semaglutide and liraglutide, respectively. Significant disproportionality was detected only for semaglutide-associated suicidal ideation (ROR, 1.45; 95% CI, 1.18-1.77; IC, 0.53; 95% CI, 0.19-0.78), which remained significant in patients with coreported use of antidepressants (ROR, 4.45; 95% CI, 2.52-7.86; IC, 1.96; 95% CI, 0.98-2.63) and benzodiazepines (ROR, 4.07; 95% CI, 1.69-9.82; IC, 1.67; 95% CI, 0.11-2.65), when compared with dapagliflozin (ROR, 5.56; 95% CI, 3.23-9.60; IC, 0.70; 95% CI, 0.36-0.95), metformin (ROR, 3.86; 95% CI, 2.91-5.12; IC, 1.20; 95% CI, 0.94-1.53) and orlistat (ROR, 4.24; 95% CI, 2.69-6.69; IC, 0.70; 95% CI, 0.36-0.95).

**Conclusions and Relevance:**

This study using the WHO database found a signal of semaglutide-associated suicidal ideation, which warrants urgent clarification.

## Key Points

**Question:**

Are glucagon-like peptide-1 receptor agonists semaglutide and liraglutide, which were originally introduced for the treatment of type 2 diabetes and are frequently prescribed due to their weight loss properties, associated with disproportionately increased reporting of suicidality?

**Findings:**

This disproportionality analysis through the case-control design based on the World Health Organization global database collecting suspected adverse drug reactions, identified a disproportionality signal of suicidal ideation with semaglutide, which remained significant when comparing semaglutide with dapagliflozin and metformin and in the subgroup of patients with coreported use of antidepressants and benzodiazepines.

**Meaning:**

A detected signal of semaglutide-associated suicidal ideation warrants urgent clarification.

## Teaser

This case control study evaluates potential signals for suicidal and self-injurious adverse drug reactions associated with semaglutide and liraglutide.

## Introduction

Over the past decade, obesity trends have reached epidemic standards.^1^ In this context, the understanding of glucagon-like peptide-1 (GLP-1)–based mechanisms and related anorectic properties of GLP-1 receptor agonists (RAs) have revolutionized the treatment of obesity.^2^ In addition to enhancing glucose-dependent insulin release, GLP-1 RAs may reduce glucagon secretion as well as gastric emptying.^2^ Originally introduced for the treatment of type 2 diabetes, the effect of GLP-1 RAs on weight loss soon caught research attention.^3^ The weight loss properties of liraglutide and semaglutide quickly went viral on social media, leveraging their promotion as lifestyle drugs not just for patients with diabetes^4^ and leading to a global shortage.^5^ Currently, it is estimated that approximately 10% of patients with type 2 diabetes in the US are prescribed GLP-1 RAs.^6^ Accordingly, regulatory authorities over the world have urged health care professionals to direct available supplies to patients with type 2 diabetes who are inadequately managed with other medications over off-label prescriptions.^7,8^ Despite the promising potential of GLP-1 RAs, serious concerns have been raised about their safety.^9,10^ On July 3, 2023, a series of reports for suicidal or self-harming thoughts associated with liraglutide or semaglutide triggered an ongoing review by the European Medicines Agency (EMA).^11^ Previously, in the approval trials, 9 of the 3384 patients treated with liraglutide (0.27%) had reported suicidal ideation compared with 2 of 1941 patients allocated to the placebo group (0.10%).^12^ On the other hand, no patients using semaglutide for obesity had developed suicidal ideation,^13,14^ and no mental health differences were observed in adolescents, with a lower percentage of participants in the semaglutide group than in the placebo group reporting psychiatric adverse events (7% vs 15%).^15^

The EMA-led investigation might have a global impact, given that liraglutide and semaglutide are administered to more than 20 million people per year.^11^ This investigation was expected to be completed in November 2023, but ultimately updated in April 2024 after requesting further clarifications.^16^ In the meantime the British Medicines and Healthcare Products Regulatory Agency and the US Food and Drug Administration (FDA) also announced a similar investigation.^17^ So far both EMA and FDA declared that they did not find any clear demonstration of a relationship between GLP-1 RAs and suicide based on the available evidence, although the FDA investigation is still ongoing.^18,19^

Marketing companies stated that warnings about suicidal behavior and ideation are formally required for medications prescribed for chronic weight management affecting the central nervous system.^20^ The first 2 pharmacovigilance studies on the topic only included partial data from the US,^21,22^ and a report from the EMA pharmacovigilance database did not assess disproportionality.^23^ Typically, patients with suicidality are excluded from clinical trials; therefore, the reports from clinical trials may be less precise in capturing the risk of suicidal or self-injurious adverse drug reactions (ADRs) in later practice. In this context, we aimed to assess suicidal and/or self-injurious ADRs associated with liraglutide or semaglutide at a global level, using a World Health Organization (WHO) database of individual case safety reports (ICSRs).

## Methods

This case-control study was conceived as a disproportionality analysis of the WHO Vigibase, a consolidated tool for postmarketing surveillance. In the past, large-scale ICSR databases attracted interest for early detection and characterization of emerging safety issues.^24,25,26^

All procedures and analyses adhered to the Uppsala Monitoring Centre (UMC) caveat agreement for reporting standards and were in accordance with the Helsinki declaration for ethical principles in medical research. As the data were anonymized and all analyses were descriptive, an ethical review from the Zurich Cantonal ethics board was not required. Additionally, per the Common Rule, because all data in the database are anonymized, patient informed consent was not required. This study was reported according to The Reporting of A Disproportionality Analysis for Drug Safety Signal Detection Using Individual Case Safety Reports in PharmacoVigilance (READUS-PV) guideline.^27,28,29^

We conducted a comprehensive search for reports of suicidal or self-injurious ADRs associated with liraglutide and semaglutide within the WHO global ICSRs database, which is the largest pharmacovigilance archive worldwide containing over 28 million reports of suspected ADRs from 140 member countries. On August 30, 2023, we selected all deduplicated ICSRs recorded in the database from inception. Reports for semaglutide were recorded between July 2011 and August 2023, whereas for liraglutide, reports were collected between November 2000 and August 2023. Database services are offered by the UMC, which manages the database.^30,31^ Drugs recorded on the reports are coded using the WHO drug dictionary^32^ and ADRs are classified according to the Medical Dictionary for Regulatory Activities (MedDRA), version 23.1.^33^ Two authors (C.G. and G.S.) identified ADRs involving liraglutide and/or semaglutide as suspected or interacting drugs to identify any report of suicidal and/or self-injurious ADRs as classified in the “suicide/self-injury” standardized MedDRA query (SMQ) of the MedDRA classification. Such SMQs include any ADR related to suicidal and/or self-injurious thoughts and events.^34^ Cases were all reports of suicidal and/or self-injurious ADRs, whereas controls were all other reports of suspected ADRs. We included liraglutide-related or semaglutide-related reports.

### Statistical Analysis

Descriptive statistics on demographic and clinical characteristics (in medians and IQRs) of reported cases were provided. We compared the percentage of female patients, age, and dose between patients prescribed GLP-1 RAs for different indications. We grouped indications into the following categories: diabetes, weight management, possible off-label indication, and others. The classification of indications is detailed in eTable 1 in Supplement 1. Comparisons of demographic and clinical characteristics were performed using χ^2^ tests, Wilcox, Kruskal-Wallis, or Fisher tests. We also explored psychiatric symptoms coreported with the suicidal and/or self-injurious ADRs of interest. Two-sided *P *values less than .05 were considered significant.

We performed disproportionality analysis using 2 consolidated measures when at least 3 reports were recorded: first, we estimated reporting odds ratio (ROR)^35^ and the bayesian information component (IC),^36^ with 95% CIs. We applied well-established thresholds to define signals of disproportionate reporting, that is, lower limit of the 95% CI greater than 1 and greater than 0 for ROR and IC, respectively. Additional details about disproportionality analysis are reported in the eMethods in Supplement 1.

For suicidal and/or self-injurious ADRs that had a signal of disproportionate reporting in the main disproportionality analysis, we performed sensitivity analyses to explore potential confounders. The following sensitivity analyses were conducted: (1) selecting only cases with coreporting of use of antidepressants and (2) only cases with coreporting of use of benzodiazepines as a proxy of depressive and anxiety disorders that can increase the risk of suicidal and/or self-injurious behaviors and ideation. Moreover, we repeated the aforementioned analyses by (3) excluding reports with coreporting of antidepressants and (4) benzodiazepines. To additionally mitigate the risk of confounding by indication and channeling bias, we performed 3 other sensitivity analyses using other drugs prescribed for the same indications (obesity and type 2 diabetes) as comparators; specifically we selected (5) dapagliflozin, a sodium-glucose cotransporter 2 inhibitor, and (6) metformin, considering their well-established role in the treatment of type 2 diabetes coupled with their favorable impact on body weight^37,38^; and (7) orlistat, considering its indication for obesity and weight loss.

We also assessed the disproportionality of reporting in female and male patients separately. Last, to assess the trend of reporting over time, we reported the number of reports by year for all ADRs of interest and for each ADR with disproportionate reporting. Data were analyzed from September to December 2023.

## Results

As of August 30, 2023, of the 36 172 078 total reports in the database, we identified a total of 107 (median [IQR] age, 48 [40-56] years; 59 female patients [55%]; median [IQR] treatment duration, 24.0 [2.3-61.0] days [data from 28 reports]) unique, deduplicated cases of suicidal and/or self-injurious ADRs associated with semaglutide (107 of the 30 527 total reports [0.35%]) and 162 (median [IQR] age, 47 [38-60] years; 100 female patients [61%]; median [IQR] treatment duration, 46.0 [14.0-99.0] days [data from 33 reports]) cases associated with liraglutide (162 of 52 131 total reports [0.31%]). Demographic and clinical characteristics of the cases are reported in Table 1 by GLP-1 RA.

**Table 1.** Demographic and Clinical Characteristics of Self-Injurious or Suicidal Adverse Drug Reactions With Semaglutide and Liraglutide

Characteristic	Patients, No. (%)	
	Semaglutide (n = 107)	Liraglutide (n = 162)
Age, median (IQR), y^a^	48 (40-56)	47 (38-60)
Sex		
Female^b^	59 (55.1)	100 (61.7)
Male	41 (38.3)	49 (30.2)
BMI, median (IQR)^c^^,^^d^	32.8 (32.4-36.4)	33.2 (24.4-38.0)
Fatal	7 (6.5)	24 (14.8)
Indications		
Possible off-label	34 (31.78)	55 (33.95)
Weight management	28 (26.17)	40 (24.70)
Diabetes	26 (24.30)	33 (20.37)
PCOS	1 (0.95)	1 (0.62)
Missing	18 (16.82)	41 (25.31)
Reporter qualification		
Consumer	52 (48.60)	51 (31.48)
Other nonhealth professional	5 (4.67)	1 (0.62)
Health professional	45 (42.06)	103 (63.58)
Unknown	5 (4.67)	7 (4.3)
Country		
Australia	9 (8.41)	21 (12.96)
North America	48 (44.86)	91 (56.17)
South America	3 (2.80)	3 (1.85)
Europe	46 (42.99)	41 (25.31)
Asia/Africa	1 (0.95)	6 (3.70)
Dose, median (IQR), mg^e^	0.50 (0.25-1.25)	1.2 (0.6-1.8)
Regimen		
Per week	34 (31.78)	NA
Per day	8 (7.48)	39 (24.07)
Missing	65 (60.75)	123 (75.93)
Action taken with suspected drug		
Drug withdrawn	35 (32.71)	66 (40.74)
Dose not changed	14 (13.09)	6 (3.70)
Dose increased	6 (5.61)	4 (2.50)
Dose reduced	1 (0.95)	2 (1.24)
Missing	51 (47.66)	84 (51.85)
Comedications		
Antidiabetics	17 (15.90)	49 (30.25)
PPIs	7 (6.54)	5 (3.09)
Statins	12 (11.22)	18 (11.11)
Analgesics	8 (7.48)	27 (16.67)
Antidepressants	14 (13.09)	30 (18.52)
Antipsychotics	3 (2.80)	13 (8.03)
Anticonvulsants	2 (1.90)	9 (5.56)
Benzodiazepines	5 (4.67)	26 (16.05)
Antihypertensives	9 (8.41)	26 (16.05)
Antiasthmatics/histaminics	5 (4.67)	2 (1.24)

Abbreviations: NA, not applicable; PCOS, polycystic ovary syndrome; PPI, proton pump inhibitor.

^a^ Data available for 73 semaglutide-related (68.2%) and 114 liraglutide-related (70.4%) cases.

^b^ Data available for 100 semaglutide-related (93.4%) and 149 liraglutide-related (92.0%) cases.

^c^ Data available for 6 semaglutide-related (5.6%) and 17 liraglutide-related (10.5%) cases.

^d^ Body mass index is calculated as weight in kilograms divided by height in meters squared.

^e^ Data available for 41 semaglutide-related (38.3%) and 48 liraglutide-related (29.6%) cases.

Regarding indications for use, the main reason for prescription was a possible off-label use (34 patients for semaglutide [31.8%] and 55 for liraglutide [33.9%]), followed by weight management (28 for semaglutide [26.2%] and 40 for liraglutide [24.7%]), diabetes (26 for semaglutide [24.3%] and 33 for liraglutide [20.4%]), and in 1 case for polycystic ovary syndrome for each GLP-1 RA (1 for semaglutide [0.9%] and 1 for liraglutide [0.6%]). Semaglutide-associated cases were reported by consumers in almost half of the reports (52 cases [48.6%]). Liraglutide-associated cases were mainly reported by health professionals (103 cases [63.6%]).

Regarding outcomes following dechallenge and rechallenge, both for semaglutide (eTable 2 in Supplement 1) and liraglutide (eTable 3 in Supplement 1), suicidal ideation resolved after drug discontinuation in 62.5% of the cases. In the semaglutide-associated reports of suicidal and/or self-injurious ADRs, the most common comedications included antidiabetics (17 patients [15.9%]) and antidepressants (14 patients [13.1%]), with higher percentages for liraglutide (49 patients [30.3%] and 30 patients [18.5%], respectively) (Table 1).

Suicidal and/or self-injurious ADRs associated with semaglutide and/or liraglutide are presented in Table 2. Suicidal ideation, intentional overdose, and suicide attempt ranked highest for semaglutide (94 patients [88%], 7 patients [6.5%], and 7 patients [6.5%], respectively), whereas for liraglutide suicidal ideation, completed suicide, and suicide attempt ranked highest (116 patients [71.6%], 19 patients [11.7%], and 16 patients [9.9%], respectively). Seven reactions (6.5%) were fatal for semaglutide and 24 (14.8%) for liraglutide. In Table 2 we report the number and percentage of each ADR by indication. For reports of suicidal ideation, we reported older age for patients prescribed liraglutide for diabetes compared with off-label and weight management, as well as a lower percentage of female patients prescribed liraglutide for diabetes compared with off-label and weight management (eTable 4 in Supplement 1).

**Table 2.** Suicidal and Self-Injurious Adverse Drug Reactions (ADRs) With Semaglutide and Liraglutide^a^

ADR	Patients, No. (%)								All medications, total No.
	Semaglutide (n = 107)				Liraglutide (n = 162)				
	Total	By indication			Total	By indication			
		Diabetes	Possible off-label	Weight management		Diabetes	Possible off-label	Weight management	
Suicidal ideation	94 (87.85)	23 (21.49)	26 (24.30)	26 (24.30)	116 (71.60)	20 (12.35)	28 (17.28)	35 (21.61)	77 266
Intentional overdose	7 (6.54)	1 (0.95)	4 (3.74)	0	4 (2.47)	0	1 (0.62)	1 (0.62)	48 096
Suicide attempt	7 (6.54)	1 (0.95)	2 (1.90)	2 (1.90)	16 (9.89)	6 (3.70)	3 (1.85)	3 (1.85)	52 024
Completed suicide	6 (5.61)	3 (2.80)	3 (2.80)	0	19 (11.73)	5 (3.09)	9 (5.56)	0	71 522
Suicidal behavior	5 (4.67)	2 (1.90)	0	0	4 (2.47)	2 (1.25)	1 (0.62)	1 (0.62)	3303
Intentional self-injury	3 (2.80)	2 (1.90)	0	1 (0.95)	4 (2.47)	0	1 (0.62)	2 (1.25)	13 019
Self-injurious ideation	3 (2.80)	1 (0.95)	0	2 (1.90)	5 (3.08)	2 (1.25)	1 (0.62)	2 (1.25)	3198
Depression suicidal	2 (1.90)	0	0	1 (0.95)	1 (0.62)	0	0	1 (0.62)	2654
Suspected suicide	0	0	0	0	5 (3.08)	0	4 (2.47)	0	2316
Cases with no coreported psychiatric symptoms	50 (47.63)	9 (8.41)	23 (21.49)	6 (5.60)	91 (56.17)	17 (10.49)	40 (24.69)	16 (9.88)	NA

Abbreviation: NA, not applicable.

^a^ Cumulatively, there were 30 527 reports of ADRs associated with semaglutide and 52 131 reports associated with liraglutide.

The list of coreported psychiatric symptoms for semaglutide and liraglutide is reported in eTable 5 and eTable 6 in Supplement 1. For liraglutide there were 91 cases in which suicidal and/or self-injurious ADRs were reported without any other psychiatric symptoms, 50 cases in which 1 psychiatric symptom was coreported, and 21 in which 2 or more other psychiatric symptoms were reported. Table 2 shows the number of cases in which suicidal and/or self-injurious ADRs were reported alone by indication. In half of these cases the drug was taken off-label both for semaglutide and liraglutide (Table 2).

### Disproportionality Analyses

We detected a significant disproportionality only for semaglutide-associated suicidal ideation compared with all medications (ROR, 1.45; 95% CI, 1.18-1.77; IC, 0.53; 95% CI, 0.19-0.78) (Table 3). Data on duration for semaglutide treatment were available in 26 patients reporting suicidal ideation, with a mean (range) duration of 80.39 (0 to 610) days between semaglutide treatment initiation and suicidal ideation occurrence. We did not find signals for any other ADR of interest (Table 3). Numbers of cases and controls are reported in eTable 7 and eTable 8 in Supplement 1.

**Table 3.** Disproportionality Analysis of Semaglutide- and Liraglutide-Associated Suicidal and Self-Injurious Adverse Drug Reactions (ADRs) Compared With All Other Drugs in the Database

ADR	Semaglutide			Liraglutide		
	No.	ROR (95% CI)	IC (95% CI)	No.	ROR (95% CI)	IC (95% CI)
Suicidal ideation	94	1.45 (1.18 to 1.77)	0.53 (0.19 to 0.78)	116	1.04 (0.87 to 1.25)	0.06 (−0.25 to 0.28)
Intentional overdose	7	0.17 (0.08 to 0.36)	−2.45 (−3.75 to −1.60)	4	0.06 (0.02 to 0.15)	−3.95 (−5.72 to −2.87)
Suicide attempt	7	0.16 (0.08 to 0.33)	−2.56 (−3.86 to −1.71)	16	0.21 (0.13 to 0.35)	−2.19 (−3.03 to −1.61)
Completed suicide	6	0.10 (0.04 to 0.22	−3.22 (−4.64 to −2.31)	19	0.18 (0.12 to 0.29)	−2.41 (−3.18 to −1.87)
Suicidal behavior	5	1.80 (0.75 to 4.32	0.75 (−0.81 to 1.73)	4	0.84 (0.32 to 2.25)	−0.22 (−1.99 to 0.86)
Intentional self-injury	3	0.27 (0.09 to 0.85	−1.71 (−3.78 to −0.50)	4	0.21 (0.08 to 0.57)	−2.09 (−3.86 to −1.01)
Self-injurious ideation	3	1.11 (0.36 to 3.46)	0.13 (−1.94 to 1.34)	5	1.09 (0.45 to 2.61)	0.11 (−1.45 to 1.09)
Suspected suicide	NA	NA	NA	5	1.5 (0.62 to 3.61)	0.52 (−1.04 to 1.5)
All ADRs combined	127^a^	0.55 (0.46 to 0.65)	−0.85 (−1.14 to −0.64)	174^a^	0.44 (0.38 to 0.51)	−1.17 (−1.42 to −0.99)

Abbreviations: IC, information component; NA, not applicable; ROR, reporting odds ratio.

^a^ Also includes 2 reports of depression suicidal for semaglutide and 1 for liraglutide, for which we did not perform disproportionality analyses (due to less than 3 reports available).

### Sensitivity Analyses

The first sensitivity analysis including cases with comedications with antidepressants showed a disproportionate reporting of semaglutide-associated suicidal ideation compared with all medications (ROR, 4.45; 95% CI, 2.52 to 7.86; IC, 1.96; 95% CI, 0.98 to 2.63). The second sensitivity analysis including cases with comedications with benzodiazepines showed a disproportionate reporting of semaglutide-associated suicidal ideation compared with all medications (ROR, 4.07; 95% CI, 1.69 to 9.82; IC, 1.67; 95% CI, 0.11 to 2.65). Reporting was not disproportionate when excluding reports with comedications with antidepressants (ROR, 1.28; 95% CI, 1.03 to 1.60; IC, 0.36; 95% CI, −0.10 to 0.63), but remained disproportionate when excluding reports with comedications with benzodiazepines (ROR, 1.40; 95% CI, 1.13 to 1.72; IC, 0.48; 95% CI, 0.13 to 0.73). The fifth sensitivity analysis showed a disproportionate reporting of semaglutide-associated suicidal ideation compared with dapagliflozin (ROR, 5.56; 95% CI, 3.23 to 9.60; IC, 0.70; 95% CI, 0.36 to 0.95). The sixth sensitivity analysis showed a disproportionate reporting of semaglutide-associated suicidal ideation compared with metformin (ROR, 3.86; 95% CI, 2.91 to 5.12; IC, 1.20; 95% CI, 0.94 to 1.53). The seventh sensitivity analysis showed a disproportionate reporting of semaglutide-associated suicidal ideation compared with orlistat (ROR, 4.24; 95% CI, 2.69 to 6.69; IC, 0.70; 95% CI, 0.36 to 0.95). The analysis of disproportionality for suicidal ideation in female and male patients separately yielded disproportionate reporting in men (ROR, 1.51; 95% CI, 1.09 to 2.08; IC, 0.58; 95% CI, 0.03 to 0.97), whereas in female patients, although the lower limit of the 95% CI of the ROR was greater than 1 (ROR, 1.35; 95% CI, 1.02 to 1.77) the lower limit of the 95% CI of the IC was less than 0 (IC, 0.42; 95% CI,−0.05 to 0.75) (eTable 9 in Supplement 1).

From marketization year till August 2023, there was a slight increase in the proportion of suicidal ADRs reported for both drugs. The increase was from 0% (2017) to 0.8% (2023) for semaglutide and from 0.09% (2014) to 0.4% (2023) for liraglutide (eTable 10 and eTable 11 in Supplement 1).

## Discussion

In this disproportionality analysis of the world’s largest ICSRs database using a case-control design, we found a significant disproportionality only for semaglutide-associated suicidal ideation compared with other medications. The number of reports showed a gradual increase over the years, which may indicate a widening therapeutic scope in obesity and accumulating clinical experience.

To our knowledge, no previous reports investigated the association between semaglutide and suicidal ideation using this database. In our sensitivity analyses, the disproportionality remained significant when focusing on coreported antidepressants or benzodiazepines, suggesting that people with anxiety and depressive disorders may be at higher probability of reporting suicidal ideation when medicated with semaglutide. When repeating the analysis after excluding cases in which antidepressants were coreported, we did not detect a disproportionality signal. In contrast, when repeating the analysis after excluding cases in which benzodiazepines were coreported, the disproportionality remained significant. This is consistent with an interaction between baseline psychopathology and semaglutide effects and warrants further investigation. Although EMA stated that no update to the product information is warranted, based on these findings, we believe that a precaution of use in patients with psychiatric disorders or psychological lability could be added in the semaglutide package insert. Remarkably, the FDA label of semaglutide for obesity warned to monitor for depression or suicidal thoughts.^39^

One study using the FDA pharmacovigilance database suggested disproportionate reporting for suicidal ideation and suicidal depression for semaglutide and liraglutide,^21^ whereas another study did not detect an association between suicidality and GLP-1 RAs.^22^ Likewise, a cohort study using electronic health records did not detect higher risks of suicidal ideation in patients with obesity or diabetes treated with semaglutide compared with non–GLP-1 RAs.^40^ Compared with this study, in our analysis we also included patients with potential off-label prescription of GLP-1 RAs. Thus, our analysis may be generalized to patients receiving GLP-1 RAs without a diagnosis of diabetes or obesity, thus further confirming the complementary nature of studies based on ICSRs disproportionality analysis and longitudinal observational design.

Evidence from bariatric studies suggests that a history of depression or anxiety is a predictor of suicide risk postoperatively, providing context for this interplay^41^; authors discussed this association in light of the emerging frustration due to high expectations of bariatric surgery outcomes in patients with limited resources to deal with mental distress.^41^ Of note, the pivotal trials of semaglutide in obesity had different exclusion criteria for mental disorders, such as major depressive disorder within 2 years before screening, diagnosis of severe psychiatric disorders, and history of suicide attempts.^13,15^ An alternative hypothesis may consider very rapid weight loss related to adjustment problems such as inability to eat as expected and ultimately exacerbated mental distress in highly vulnerable patients.^41^ Due to lack of data on GLP-1 RA–related changes of baseline weight or BMI, we could not test for either hypothesis.

Although comorbidity with depression is high in patients with diabetes,^42^ the coreporting between antidepressants and antidiabetics was negligible. Furthermore, the signal of semaglutide remained when comparing with dapagliflozin, metformin, and orlistat, mitigating the risk of confounding by indication for diabetes and obesity. Therefore, patients with diabetes and/or obesity without psychiatric comorbidities may not be at high risk of semaglutide-associated suicidal ideation. Although ADR incidence cannot be calculated using the spontaneous reporting system, this ADR is likely to be rare and would probably not substantially alter the benefit-risk profile of semaglutide in approved therapeutic settings. However, the observed high proportion of cases due to possible off-label use and a recently published postmarketing signal of misuse or abuse^43^ call for urgent clarification of patient-related and drug-related risk factors; cohort studies and large registries should be stratified by therapeutic indication, sex, and history of mental disorders, and data from off-label use should be retrieved.

Despite the large number of cases, we did not detect any signals for liraglutide-associated suicidal and/or self-injurious ADRs. Pooled data from phase 2 and 3 trials on liraglutide vs placebo for weight management identified a potential risk for suicidal ideation.^12^ Nine of 3384 participants in the liraglutide group vs 2 of 1941 in the placebo group reported suicidal ideation or behavior during the trial (0.27% vs 0.10%).^12^

### Limitations

The results of this study should be interpreted in light of several limitations.^25^ First, we need to consider barriers to reporting and missing information. Second, the well-known inability to infer causality does not allow us to attribute any reactions to the effect of a drug. Third, the lack of denominator does not allow us to estimate the incidence of ADRs. Fourth, selection and collider bias,^44^ as well as confounding by indication and channeling bias, although partially mitigated by our sensitivity analyses, may have played a role as people with treatment-resistant diabetes or obesity might reflect a subgroup of patients with more severe conditions including higher risk of mental distress. Additional adjustments for potential confounders, such as alcohol or substance misuse, were limited by the relatively small number of reports found. In the absence of more details about off-label prescribing, we were not able to further qualify the extent to which prescribing was off-label and its impact on the results. Fifth, the lack of treatment outcomes, such as weight change, did not allow different hypotheses to be considered. Sixth, the high proportion of cases with missing data on medication dose precluded a dose-response analysis. Seventh, because of the absence of information on the sociodemographic profile of the reporters, it is impossible to account for volunteer bias. Eighth, it is not possible to exclude the chance that, for instance, suicidal ideation may have preexisted. Ninth, data on treatment duration until ADR were provided only in a small number of reports. Additionally, since disproportionality measures are interdependent, the lack of statistically significant disproportionality should not be automatically interpreted as a safety endorsement. Several factors may influence the reporting pattern and the ability to detect disproportionality, including known and widely reported ADRs such as gastrointestinal ADRs.

Our findings are relevant to the general reader seeking up-to-date information. This relevance arises from the expectation that personal or anecdotal reports may continue to gain popularity on social media platforms without knowledge about risks.^45^ One consequence of this trend may be the increase in off-label use of semaglutide, which is a public health concern that has led to the illegal trade in semaglutide pens, some of which are counterfeit.^46^ Recently, a public warning about fake counterfeit semaglutide pens was issued in the United Kingdom and the US.^46,47^ Considering the risk of suicidal ideation in people taking semaglutide off-label, authorities should consider issuing a warning to inform about this risk.

## Conclusions

In this disproportionality study of an ADR database, we reported a disproportionality signal of suicidal ideation with semaglutide, but not for liraglutide, particularly among patients with coreported antidepressant use, a proxy for affective disorders (a notable exclusion criteria of premarketing clinical trials).

## Notes

Supplement 1. **eTable 1.** Classification of Indications
eMethods.
**eTable 2.** Dechallenge and Rechallenge With Semaglutide **eTable 3. **Dechallenge and Rechallenge With Liraglutide **eTable 4.** Sex, Median Age and Dose (IQR) for Suicidal Ideation ADRs by Drug and Indication **eTable 5.** Coreported Psychiatric Reactions for Semaglutide **eTable 6.** Coreported Psychiatric Reactions for Liraglutide by Indication **eTable 7.** Number of Cases, Noncases, Other Adverse Drug Reactions (ADRs) and Total Number of Other Reports in the Database for Semaglutide **eTable 8. **Number of Cases, Noncases, Other Adverse Drug Reactions (ADRs) and Total Number of Other Reports in the Database for Liraglutide **eTable 9.** Disproportionality Analysis of Semaglutide-Associated Suicidal Ideation Compared With All Other Drugs in the Database in Female and Male Patients Separately **eTable 10.** Number of Semaglutide-Associated Cases of Adverse Drug Reactions (ADRs) by Year **eTable 11.** Number of Liraglutide-Associated Cases of Adverse Drug Reactions (ADRs) by Year
eReferences.

Supplement 2.
Data Sharing Statement

## References

[1] Collaboration NCDRF; NCD Risk Factor Collaboration (NCD-RisC). Trends in adult body-mass index in 200 countries from 1975 to 2014: a pooled analysis of 1698 population-based measurement studies with 19·2 million participants. Lancet. 2016;387(10026):1377-1396. doi:10.1016/S0140-6736(16)30054-X 27115820 PMC7615134

[2] Drucker DJ. GLP-1 physiology informs the pharmacotherapy of obesity. Mol Metab. 2022;57:101351. doi:10.1016/j.molmet.2021.101351 34626851 PMC8859548

[3] Fonseca VA, Capehorn MS, Garg SK, . Reductions in insulin resistance are mediated primarily via weight loss in subjects with type 2 diabetes on semaglutide. J Clin Endocrinol Metab. 2019;104(9):4078-4086. doi:10.1210/jc.2018-02685 30938762

[4] CBS News. Weight loss TikTok trend triggers shortage of diabetic medication. Accessed February 16, 2024. https://www.cbsnews.com/baltimore/news/weight-loss-tiktok-trend-triggers-shortage-of-diabetic-medication/

[5] US Food and Drug Administration. FDA drug shortages. Accessed February 16, 2024. https://www.accessdata.fda.gov/scripts/drugshortages/

[6] Eberly LA, Yang L, Essien UR, . Racial, ethnic, and socioeconomic inequities in glucagon-like peptide-1 receptor agonist use among patients with diabetes in the US. JAMA Health Forum. 2021;2(12):e214182. doi:10.1001/jamahealthforum.2021.4182 35977298 PMC8796881

[7] Australian Goverment Department of Health and Aged Care Therapeutic Goods Administration. About the Ozempic (semaglutide) shortage 2022 and 2023. Accessed February 16, 2024. https://www.tga.gov.au/safety/shortages/information-about-major-medicine-shortages/about-ozempic-semaglutide-shortage-2022-and-2023

[8] Iacobucci G. UK clinics told to stop prescribing antidiabetes drugs for weight loss, after shortages. BMJ. 2023;382:1693. doi:10.1136/bmj.p1693 37479232

[9] Abrahami D, Douros A, Yin H, . Incretin based drugs and risk of cholangiocarcinoma among patients with type 2 diabetes: population based cohort study. BMJ. 2018;363:k4880. doi:10.1136/bmj.k4880 30518618 PMC6278586

[10] Bezin J, Gouverneur A, Pénichon M, . GLP-1 receptor agonists and the risk of thyroid cancer. Diabetes Care. 2023;46(2):384-390. doi:10.2337/dc22-1148 36356111

[11] European Medicines Agency. EMA statement on ongoing review of GLP-1 receptor agonists. Accessed September 4, 2023. https://www.ema.europa.eu/en/news/ema-statement-ongoing-review-glp-1-receptor-agonists

[12] O’Neil PM, Aroda VR, Astrup A, ; Satiety and Clinical Adiposity - Liraglutide Evidence in individuals with and without diabetes (SCALE) study groups. Neuropsychiatric safety with liraglutide 3.0 mg for weight management: Results from randomized controlled phase 2 and 3a trials. Diabetes Obes Metab. 2017;19(11):1529-1536. doi:10.1111/dom.12963 28386912 PMC5655710

[13] Wilding JPH, Batterham RL, Calanna S, ; STEP 1 Study Group. Once-weekly semaglutide in adults with overweight or obesity. N Engl J Med. 2021;384(11):989-1002. doi:10.1056/NEJMoa2032183 33567185

[14] Andreadis P, Karagiannis T, Malandris K, . Semaglutide for type 2 diabetes mellitus: a systematic review and meta-analysis. Diabetes Obes Metab. 2018;20(9):2255-2263. doi:10.1111/dom.13361 29756388

[15] Weghuber D, Barrett T, Barrientos-Pérez M, ; STEP TEENS Investigators. Once-weekly semaglutide in adolescents with obesity. N Engl J Med. 2022;387(24):2245-2257. doi:10.1056/NEJMoa2208601 36322838 PMC9997064

[16] European Medicines Agency. GLP-1 receptor agonists’ review: PRAC requests further clarifications from marketing authorisation holders. Accessed February 16, 2024. https://www.ema.europa.eu/en/news/meeting-highlights-pharmacovigilance-risk-assessment-committee-prac-27-30-november-2023

[17] Reuters. UK probing novos ozembic weight loss drug saxenda over suicidal self-harming. Accessed September 4, 2023. https://www.reuters.com/business/healthcare-pharmaceuticals/uk-probing-novos-ozempic-weight-loss-drug-saxenda-over-suicidal-self-harming-2023-07-26/

[18] US Food and Drug Administration. Update on FDA’s ongoing evaluation of reports of suicidal thoughts or actions in patients taking a certain type of medicines approved for type 2 diabetes and obesity. Preliminary evaluation does not suggest a causal link. Accessed February 16, 2024. https://www.fda.gov/drugs/drug-safety-and-availability/update-fdas-ongoing-evaluation-reports-suicidal-thoughts-or-actions-patients-taking-certain-type

[19] European Medicines Agency. Meeting highlights from the Pharmacovigilance Risk Assessment Committee (PRAC) 8-11 April 2024. Accessed May 19, 2024. https://www.ema.europa.eu/en/news/meeting-highlights-pharmacovigilance-risk-assessment-committee-prac-8-11-april-2024

[20] Youmshajekian L. Evidence weighed for suicide/self-harm with obesity drugs. Accessed September 4, 2023. https://www.medscape.com/viewarticle/994266?form=fpf

[21] McIntyre RS, Mansur RB, Rosenblat JD, Kwan ATH. The association between glucagon-like peptide-1 receptor agonists (GLP-1 RAs) and suicidality: reports to the Food and Drug Administration Adverse Event Reporting System (FAERS). Expert Opin Drug Saf. 2024;23(1):47-55. doi:10.1080/14740338.2023.2295397 38087976

[22] Chen C, Zhou R, Fu F, Xiao J. Postmarket safety profile of suicide/self-injury for GLP-1 receptor agonist: a real-world pharmacovigilance analysis. Eur Psychiatry. 2023;66(1):e99. doi:10.1192/j.eurpsy.2023.2474 38031404 PMC10755578

[23] Tobaiqy M, Elkout H. Psychiatric adverse events associated with semaglutide, liraglutide and tirzepatide: a pharmacovigilance analysis of individual case safety reports submitted to the EudraVigilance database. Int J Clin Pharm. 2024;46(2):488-495. doi:10.1007/s11096-023-01694-7 38265519 PMC10960895

[24] Cepaityte D, Siafis S, Papazisis G. Safety of antipsychotic drugs: a systematic review of disproportionality analysis studies. Behav Brain Res. 2021;404:113168. doi:10.1016/j.bbr.2021.113168 33581145

[25] Raschi E, Poluzzi E, Salvo F, . Pharmacovigilance of sodium-glucose co-transporter-2 inhibitors: what a clinician should know on disproportionality analysis of spontaneous reporting systems. Nutr Metab Cardiovasc Dis. 2018;28(6):533-542. doi:10.1016/j.numecd.2018.02.014 29625780

[26] Sartori D, Aronson JK, Norén GN, Onakpoya IJ. Signals of adverse drug reactions communicated by pharmacovigilance stakeholders: a scoping review of the global literature. Drug Saf. 2023;46(2):109-120. doi:10.1007/s40264-022-01258-0 36469249 PMC9883307

[27] READUS-PV. The REporting of A Disproportionality analysis for drUg Safety signal detection using individual case safety reports in PharmacoVigilance. Accessed May 17, 2024. https://readus-statement.org/10.1007/s40264-024-01421-9 PMC11116242 38713346

[28] Fusaroli M, Salvo F, Begaud B, . The REporting of A Disproportionality Analysis for DrUg Safety Signal Detection Using Individual Case Safety Reports in PharmacoVigilance (READUS-PV): explanation and elaboration. Drug Saf. 2024;47(6):585-599. doi:10.1007/s40264-024-01423-7 38713347 PMC11116264

[29] Fusaroli M, Salvo F, Begaud B, . The Reporting of a Disproportionality Analysis for Drug Safety Signal Detection Using Individual Case Safety Reports in PharmacoVigilance (READUS-PV): development and statement. Drug Saf. 2024;47(6):575-584. doi:10.1007/s40264-024-01421-9 38713346 PMC11116242

[30] Lindquist M. VigiBase, the WHO Global ICSR database system: basic facts. Drug Inf J. 2008;42(5):409-419. doi:10.1177/009286150804200501

[31] Uppsala Monitoring center. Accessed February 2021. https://who-umc.org/vigibase/

[32] World Health Organization. ATC/DDD index. Accessed January 20, 2024. https://www.whocc.no/atc_ddd_index/

[33] World Health Organization. MedDRA hierarchy. Accessed January 20, 2024. https://www.meddra.org/how-to-use/basics/hierarchy

[34] Salvo F, Micoulaud-Franchi JA, Palagini L, Geoffroy PA. Dual orexin receptor antagonists and suicide risk: findings from the WHO spontaneous reporting database. J Clin Psychiatry. 2023;84(6):23br14923. doi:10.4088/JCP.23br14923 37756127

[35] van Puijenbroek EP, Bate A, Leufkens HG, Lindquist M, Orre R, Egberts AC. A comparison of measures of disproportionality for signal detection in spontaneous reporting systems for adverse drug reactions. Pharmacoepidemiol Drug Saf. 2002;11(1):3-10. doi:10.1002/pds.668 11998548

[36] Bate A, Lindquist M, Edwards IR, . A bayesian neural network method for adverse drug reaction signal generation. Eur J Clin Pharmacol. 1998;54(4):315-321. doi:10.1007/s002280050466 9696956

[37] Pu R, Shi D, Gan T, . Effects of metformin in obesity treatment in different populations: a meta-analysis. Ther Adv Endocrinol Metab. Published online May 21, 2020. doi:10.1177/2042018820926000 32499908 PMC7243386

[38] ElSayed NA, Aleppo G, Aroda VR, ; on behalf of the American Diabetes Association. 9. Pharmacologic approaches to glycemic treatment: standards of care in diabetes-2023. Diabetes Care. 2023;46(suppl 1):S140-S157. doi:10.2337/dc23-S009 36507650 PMC9810476

[39] Novo Nordisk. WEGOVY (semaglutide) injection, for subcutaneous use. Accessed July 31, 2024. https://www.accessdata.fda.gov/drugsatfda_docs/label/2024/215256s011lbl.pdf

[40] Wang W, Volkow ND, Berger NA, Davis PB, Kaelber DC, Xu R. Association of semaglutide with risk of suicidal ideation in a real-world cohort. Nat Med. 2024;30(1):168-176. doi:10.1038/s41591-023-02672-2 38182782 PMC11034947

[41] Kauppila JH, Santoni G, Tao W, . Risk factors for suicide after bariatric surgery in a population-based nationwide study in five Nordic countries. Ann Surg. 2022;275(2):e410-e414. doi:10.1097/SLA.0000000000004232 32657942

[42] Waitzfelder B, Gerzoff RB, Karter AJ, . Correlates of depression among people with diabetes: the Translating Research Into Action for Diabetes (TRIAD) study. Prim Care Diabetes. 2010;4(4):215-222. doi:10.1016/j.pcd.2010.07.002 20832375 PMC4269468

[43] Chiappini S, Vickers-Smith R, Harris D, . Is there a risk for semaglutide misuse? Focus on the Food and Drug Administration’s FDA Adverse Events Reporting System (FAERS) pharmacovigilance dataset. Pharmaceuticals (Basel). 2023;16(7):994. doi:10.3390/ph16070994 37513906 PMC10384093

[44] Elwert F, Winship C. Endogenous selection bias: the problem of conditioning on a collider variable. Annu Rev Sociol. 2014;40:31-53. doi:10.1146/annurev-soc-071913-043455 30111904 PMC6089543

[45] Bremmer MP, Hendershot CS. Social media as pharmacovigilance: the potential for patient reports to inform clinical research on glucagon-like peptide 1 (GLP-1) receptor agonists for substance use disorders. J Stud Alcohol Drugs. 2023. doi:10.15288/jsad.23-00318 37917019 PMC10846600

[46] US Food and Drug Administration. Medications containing semaglutide marketed for type 2 diabetes or weight loss. Accessed February 16, 2024. https://www.fda.gov/drugs/postmarket-drug-safety-information-patients-and-providers/medications-containing-semaglutide-marketed-type-2-diabetes-or-weight-loss

[47] Sample I. UK public warned over dangers of fake weight loss medication pens. Pre-filled injection devices claimed to hold Ozempic or Saxenda may contain other substances, regulator says. The Guardian. Accessed August 11, 2023. https://www.theguardian.com/society/2023/oct/26/uk-public-warned-over-dangers-of-fake-weight-loss-medication-pens

