# Supplementary material for: Disproportionality Analysis From World Health Organization Data on Semaglutide, Liraglutide, and Suicidality
Source: JAMA Netw Open. 2024 Aug 20;7(8):e2423385. doi: 10.1001/jamanetworkopen.2024.23385 (PMC11337067; doi:10.1001/jamanetworkopen.2024.23385)
Supplement: Supplement 1. — eTable 1. Classification of Indications eMethods. eTable 2. Dechallenge and Rechallenge With Semaglutide eTable 3. Dechallenge and Rechallenge With Liraglutide eTable 4. Sex, Median Age and Dose (IQR) for Suicidal Ideation ADRs by Drug and Indication eTable 5. Coreported Psychiatric Reactions for Semaglutide eTable 6. Coreported Psychiatric Reactions for Liraglutide by Indication eTable 7. Number of Cases, Noncases, Other Adverse Drug Reactions (ADRs) and Total Number of Other Reports in the Database for Semaglutide eTable 8. Number of Cases, Noncases, Other Adverse Drug Reactions (ADRs) and Total Number of Other Reports in the Database for Liraglutide eTable 9. Disproportionality Analysis of Semaglutide-Associated Suicidal Ideation Compared With All Other Drugs in the Database in Female and Male Patients Separately eTable 10. Number of Semaglutide-Associated Cases of Adverse Drug Reactions (ADRs) by Year eTable 11. Number of Liraglutide-Associated Cases of Adverse Drug Reactions (ADRs) by Year eReferences. [file jamanetwopen-e2423385-s001.pdf]

Supplemental Online Content

Schoretsanitis G, Weiler S, Barbui C, Raschi E, and Gastaldon C. Disproportionality analysis from data on semaglutide, liraglutide, and suicidality. *JAMA Netw Open*. 2024;7(7):e2423385. doi:10.1001/jamanetworkopen.2024.23385

eTable 1. Classification of Indications

eMethods

eTable 2. Dechallenge and Rechallenge With Semaglutide

eTable 3. Dechallenge and Rechallenge With Liraglutide

eTable 4. Sex, Median Age and Dose (IQR) for Suicidal Ideation ADRs by Drug and Indication

eTable 5. Coreported Psychiatric Reactions for Semaglutide

eTable 6. Coreported Psychiatric Reactions for Liraglutide by Indication

eTable 7. Number of Cases, Noncases, Other Adverse Drug Reactions (ADRs) and Total Number of Other Reports in the Database for Semaglutide

eTable 8. Number of Cases, Noncases, Other Adverse Drug Reactions (ADRs) and Total Number of Other Reports in the Database for Liraglutide

eTable 9. Disproportionality Analysis of Semaglutide-Associated Suicidal Ideation Compared With All Other Drugs in the Database in Female and Male Patients Separately

eTable 10. Number of Semaglutide-Associated Cases of Adverse Drug Reactions (ADRs) by Year

eTable 11. Number of Liraglutide-Associated Cases of Adverse Drug Reactions (ADRs) by Year

eReferences

This supplemental material has been provided by the authors to give readers additional information about their work.

© 2024 Schoretsanitis et al. *JAMA Network Open.*

eTable 1. Classification of Indications

New category	Indications reported in the Vigibase®
Weight management	"Weight control", Obesity", "Overweight", "Weight decreased", "Adipositas", "Inability to lose weight", "Metabolic syndrome", Morbid obesity", "Body mass index high"
Possible Off-label use	"Product used for unknown indication", "Drug use for unknown indication"
Diabetes	"Type 2 diabetes mellitus", "Diabetes mellitus", "Diabetes", "Type II diabetes mellitus", "Type II diabetes mellitus inadequate control", "Blood glucose increased", "Glucose tolerance impaired", "Glycosylated haemoglobin abnormal", "Blood glucose increased"
Others	Any other indication

eMethods

We performed two different disproportionality approaches to increase consistency and robustness of results and reduce the risk of finding false positive signals.

We estimated the reporting odds ratio (ROR) and the Bayesian information component (IC)[1] for all adverse drug reactions (ADRs) with at least four reports using the R packages PhViD and BCPNN.

The ROR is calculated as the ratio of the odds of reporting a specific ADR for a particular drug (case) compared to the odds of reporting the same event for all other drugs in the database (non-cases),[2-4] similarly to the calculation of an odds ratio (OR) of a case-control study. The formula for the ROR is:

$$ROR = \frac{a/b}{c/d}$$

Where a is the number of reports for the specific ADR and the specific drug; b is the total number of reports for the specific drug, excluding the specific ADR; c is the number of reports for the specific AE and all other drugs and d is the total number of reports for all other drugs, excluding the specific ADR. Interpretations and threshold of RORs is the same of odds ratios for case-control studies, so a disproportionality is detected when the lower limit of the 95% confidence interval of ROR>1.[4]

The ROR (also known as frequentist method) is generally employed as it is relatively easy to understand, interpret and compute for clinicians. This statistical measure expresses the extent to which the reported ADR is associated with the suspected drug compared with the other drugs in the database. The occurrence of ADRs related to other drugs in the database is used as a proxy for the background incidence of ADRs (the denominator is unknown in pharmacovigilance).

Conversely, IC is a Bayesian method based on Bayes' law to estimate the probability (posterior probability) that the suspected event occurs given the use of suspect drug. Specifically, the IC is a shrinkage-based measure of observed-to-expected ratios[5] and it is computed as:

$$IC = \log_2 \frac{O + 0.5}{E + 0.5}$$

where O and E are the observed and expected numbers, respectively, of reports on the drug–ADR pair. E is given by (N A × N D)/N, where N A is the total number of reports on the ADR; N D is the total number of reports on the drug; and N is the total number of reports.[6] Credibility intervals for the IC are obtained via the Gamma distribution, and IC_{α} denotes the α percentile of the posterior distribution for the IC.[6] A signal of disproportionate reporting is detected when the lower limit of the 95% credibility interval is >0.[1] Higher ROR/IC estimates reflect stronger disproportion although there is no direct correlation between the extent of disproportionality and the strength of the drug-event association.

Methodological studies compared the performance of various approaches and suggested that sensitivity, specificity, and early identification of safety issues are largely comparable among

© 2024 Schoretsanitis et al. *JAMA Network Open.*

disproportionality measures, especially when the number of cases for ADRs considered is more than three. In case of larger level of confounding is expected/anticipated and/or the effect sizes increases, Bayesian approaches may be preferred.[7] Conversely, the ROR the has the tendency to inflate estimates in the setting of rare adverse events. To increase the robustness of our findings and mitigate the risk of identifying false positive drug–event associations, especially in the context of ADRs characterized by low or rare incidence with a probable drug-attributable component, employing multiple disproportionality approaches is deemed advantageous. Consequently, we employed two distinct approaches for signal detection. Our approach reinforces the credibility of the disproportionality signals identified, thereby contributing to the validity of our study.

© 2024 Schoretsanitis et al. *JAMA Network Open.*

The REporting of A Disproportionality analysis for drUg Safety signal detection using individual case safety reports in PharmacoVigilance (READUS-PV) Checklist

© 2024 Schoretsanitis et al. *JAMA Network Open.*

eTable 2. Dechallenge and Rechallenge With Semaglutide

**Action**		**Suicidal ideation**		**Intentional overdose**		**Suicidal behavior**		**Suicide attempt**		**Intentional self-injury**		**Self-injurious ideation**		**Depression suicidal**	
**Dechallenge**		Yes	No	Yes	No	Yes	No	Yes	No	Yes	No	Yes	No	Yes	No
		40	12		1	1	2	1	1	1	1		1	1	
Resolved after action	Yes	25	3		1	1		1						1	
	No	4	8				1								
	Unknown	10	1				1		1	1	1		1		
	NA	1													
**Rechallenge**		Yes	No	Yes	No	Yes	No	Yes	No	Yes	No	Yes	No	Yes	No
		24	-	4		5		3		1				2	
Resolved after action	Yes														
	No														
	Unknown	24		4		5		3		1				2	
	NA														

NA= not available

© 2024 Schoretsanitis et al. *JAMA Network Open.*

eTable 3. Dechallenge and Rechallenge With Liraglutide

**ADR**		**Suicidal ideation**		**Intentional overdose**		**Suicidal behaviour**		**Suicide attempt**		**Intentional self-injury**		**Self-injurious ideation**		**Depression suicidal**	
**Dechallenge**		Yes	No	Yes	No	Yes	No	Yes	No	Yes	No	Yes	No	Yes	No
		60	3	1		3		5	1			2		1	
Resolved after action	Yes	42	1	1		3		4	1			2			
	No	5	2											1	
	Unknown	11						1							
	NA	2													
**Rechallenge**		Yes	No	Yes	No	Yes	No	Yes	No	Yes	No	Yes	No	Yes	No
		35		1		1		6		2		4			
Resolved after action	Yes	1													
	No	1													
	Unknown			1		1		6		2		4			
	NA														

ADR= adverse drug reaction, NA= not available

eTable 4. Sex, Median Age and Dose (IQR) for Suicidal Ideation ADRs by Drug and Indication.

	**Semaglutide**			**Liraglutide**		
**Sex**	Female	Male	p-val*	Female	Male	p-val*
Diabetes	10	13	.20 (df=2)	10	10	**.02** (df=2)
Possible Off-label	10	13		23	4	
Weight management	17	9		28	7	
**Age, median [IQR], years**			p-val**	**Age, median [IQR], years**		p-val**
Diabetes	52.0 [41.5-59.5]		H(2)=2.63, *p*=.27	60.0 [52.2-64]		H(2)=15.30, **p<.001**
Possible Off-label	50.0 [42.5-58.0]			43.0 [39.0-59.0]		
Weight management	48.0 [34.0-53.0]			38.0 [34.0-49.0]		
**Dose, median [IQR], mg**			p-val**	**Dose, median [IQR], mg**		p-val**
Diabetes	0.5 [0.44-1.0]		H(2)=1.29, *p*=.52	1.1 [1.0-1.2]		H(2)=5.46, *p*=.06
Possible Off-label	0.5 [0.25-0.5]			2.4 [2.1-2.7]		
Weight management	0.5 [0.25-5.0]			1.2 [0.9-1.5]		

ADRs= adverse drug reactions, df= degrees of freedom, IQR= interquartile range
*Comparisons of sex distributions by indication were performed using the Fisher's Exact test.
**Comparisons of age and dose by indication were performed using the Kruskal-Wallis test.

© 2024 Schoretsanitis et al. *JAMA Network Open.*

eTable 5. Coreported Psychiatric Reactions for Semaglutide.

	No.
Depression	33
Anxiety	13
Depressed mood	11
Apathy	3
Paranoia	3
Crying	2
Disturbance in attention	2
Feeling abnormal	2
Feeling of despair	2
Insomnia	2
Irritability	2
Negative thoughts	2
Nightmare	2
Obsessive thoughts	2
Panic attack	2
Restlessness	2
Abnormal behaviour	1
Abnormal dreams	1
Abulia	1
Depersonalisation/derealisation disorder	1
Emotional distress	1
Fear	1
Frustration tolerance decreased	1
Hallucination	1
Intrusive thoughts	1
Judgement impaired	1
Mental disorder	1
Middle insomnia	1
Mood swings	1
Panic reaction	1
Personality change	1
Psychiatric symptom	1
Psychotic disorder	1
Sleep disorder	1
Stress	1
Tearfulness	1

© 2024 Schoretsanitis et al. *JAMA Network Open.*

eTable 6. Coreported Psychiatric Reactions for Liraglutide by Indication

	No.
Depression	41
Anxiety	11
Crying	7
Depressed mood	7
Mood altered	5
Major depression	3
Mental disorder	3
Social avoidant behaviour	3
Affective disorder	2
Insomnia	2
Intrusive thoughts	2
Nervousness	2
Abnormal dreams	1
Apathy	1
Bipolar I disorder	1
Confusional state	1
Depressive symptom	1
Dysphoria	1
Emotional disorder	1
Feeling abnormal	1
Hallucination	1
Homicidal ideation	1
Mood swings	1
Paranoia	1
Psychotic disorder	1
Thinking abnormal	1

© 2024 Schoretsanitis et al. *JAMA Network Open.*

eTable 7. Number of Cases, Noncases, Other Adverse Drug Reactions (ADRs) and Total Number of Other Reports in the Database for Semaglutide

ADRs	Cases	Non-cases	No. of other ADRs	Total number of other reports (excluding the ADR of interest)
Suicidal ideation	94	30 433	77 057	36 095 021
Intentional overdose	7	30 520	48 067	36 124 011
Suicide attempt	7	30 520	52 002	36 120 076
Completed suicide	6	30 521	71 497	36 100 581
Suicidal behaviour	5	30 522	3 294	36 168 784
Intentional self-injury	3	30 524	13 012	36 159 066
Self-injurious ideation	3	30 524	3 190	36 168 888

ADR=Adverse drug reaction

eTable 8. Number of Cases, Noncases, Other Adverse Drug Reactions (ADRs) and Total Number of Other Reports in the Database for Liraglutide

ADRs	Cases	Non-cases	No. of other ADRs	Total number of other reports (excluding the ADR of interest)
Suicidal ideation	116	52 015	77 057	36 095 021
Intentional overdose	4	52 127	48 067	36 124 011
Suicide attempt	16	52 115	52 002	36 120 076
Completed suicide	19	52 112	71 497	36 100 581
Suicidal behaviour	4	52 127	3 294	36 168 784
Intentional self-injury	4	52 127	13 012	36 159 066
Self-injurious ideation	5	52 126	3 190	36 168 888
Suspected suicide	5	52 126	2 311	36 169 767

ADR= adverse drug reaction

eTable 9. Disproportionality Analysis of Semaglutide-Associated Suicidal Ideation Compared With All Other Drugs in the Database in Female and Male Patients Separately.

	Semaglutide						
	No.	ROR	Lower 95%CI	Higher 95%CI	IC	Lower 95%CI	Higher 95%CI
Women	31	0.82	0.58	1.17	-0.29	-0.89	0.13
Men	19	0.77	0.49	1.21	-0.36	-1.13	0.18

© 2024 Schoretsanitis et al. *JAMA Network Open.*

CI= confidence interval, IC= information component, ROR= reporting odds ratio

eTable 10. Number of Semaglutide-Associated Cases of Adverse Drug Reactions (ADRs) by Year

Year	Suicidal ideation	All suicidal ADRs	All ADRs	Percentage of suicidal ideation/all ADRs	Percentage of all suicidal ADRs /all ADRs
2023	104	121	12 504	0.83%	0.97%
2022	14	15	9 179	0.15%	0.16%
2021	11	12	6 063	0.18%	0.20%
2020	3	4	4 515	0.07%	0.09%
2019	4	4	2 892	0.14%	0.14%
2018	0	0	189	0%	0%
2017	0	0	8	0%	0%

ADR= adverse drug reaction

eTable 11. Number of Liraglutide-Associated Cases of Adverse Drug Reactions (ADRs) by Year.

Year	Suicidal ideation	All suicidal ADRs	All ADRs	Percentage of suicidal ideation/all ADRs	Percentage of all suicidal ADRs /all ADRs
2023	33	44	7 637	0.43%	0.58%
2022	15	15	5 863	0.26%	0.26%
2021	6	6	4079	0.15%	0.15%
2020	7	20	3 405	0.21%	0.59%
2019	13	15	4 185	0.31%	0.36%
2018	10	14	3 759	0.27%	0.37%
2017	4	7	4 111	0.10%	0.17%
2016	12	17	1 870	0.64%	0.91%
2015	6	10	4 616	0.13%	0.22%
2014	4	6	4 296	0.09%	0.14%

ADR= adverse drug reaction

eReferences

1. Bate A, Lindquist M, Edwards IR, et al. A Bayesian neural network method for adverse drug reaction signal generation. *Eur J Clin Pharmacol*. 1998;54(4):315-21.
2. Pace ND, Multani JK. On the Reporting of Odds Ratios and Risk Ratios. *Nutrients*. 2018;10(10)
3. Rothman KJ, Lanes S, Sacks ST. The reporting odds ratio and its advantages over the proportional reporting ratio. *Pharmacoepidemiol Drug Saf*. 2004;13(8):519-23.
4. van Puijenbroek EP, Bate A, Leufkens HG, Lindquist M, Orre R, Egberts AC. A comparison of measures of disproportionality for signal detection in spontaneous reporting systems for adverse drug reactions. *Pharmacoepidemiol Drug Saf*. 2002;11(1):3-10.
5. Caster O, Juhlin K, Watson S, Noren GN. Improved statistical signal detection in pharmacovigilance by combining multiple strength-of-evidence aspects in vigiRank. *Drug saf*. 2014;37(8):617-28.
6. Noren GN, Hopstadius J, Bate A. Shrinkage observed-to-expected ratios for robust and transparent large-scale pattern discovery. *Stat Methods Med Res*. 2013;22(1):57-69.
7. Dijkstra L, Garling M, Foraita R, Pigeot I. Adverse drug reaction or innocent bystander? A systematic comparison of statistical discovery methods for spontaneous reporting systems. *Pharmacoepidemiol Drug Saf*. 2020;29(4):396-403.

© 2024 Schoretsanitis et al. *JAMA Network Open.*
