# Supplementary material for: Disproportionality Analysis From World Health Organization Data on Semaglutide, Liraglutide, and Suicidality
Source: JAMA Netw Open. 2024 Aug 20;7(8):e2423385. doi: 10.1001/jamanetworkopen.2024.23385 (PMC11337067; doi:10.1001/jamanetworkopen.2024.23385)
Supplement: Supplement 2. — Data Sharing Statement [file jamanetwopen-e2423385-s002.pdf]

Data Sharing Statement

Schoretsanitis. Disproportionality Analysis From Data on Semaglutide, Liraglutide, and Suicidality. *JAMA Netw Open*. Published July 30, 2024.
doi:10.1001/jamanetworkopen.2024.23385

Data
Data available: No

Additional Information
Explanation for why data not available: Vigibase® does not allow the distribution of the file, but the tables and supplementary material have all detailed information needed to perform the analyses. Other requests for data can be submitted to the UMC. The code is available at https://github.com/chiaragastaldon/semaglutide-liraglutide_pharmacovigilance-analysis.
